# Epidemiology of Surgical Site Infections With Staphylococcus aureus in Europe: Protocol for a Retrospective, Multicenter Study

**DOI:** 10.2196/resprot.8177

**Published:** 2018-03-12

**Authors:** Sibylle C Mellinghoff, Jörg Janne Vehreschild, Blasius J Liss, Oliver A Cornely

**Affiliations:** 1 Department I of Internal Medicine University Hospital of Cologne University of Cologne Cologne Germany; 2 Department I of Internal Medicine Helios University Hospital Wuppertal Wuppertal Germany; 3 Cluster of Excellence Cellular Stress Responses in Aging-Associated Diseases University of Cologne Cologne Germany

**Keywords:** surgical site infection, *Staphylococcus aureus*, cohort study, nested case-control

## Abstract

**Background:**

Surgical site infections (SSIs) are among the most common hospital acquired infections. While the incidence of SSI in certain indicator procedures is the subject of ongoing surveillance efforts in hospitals and health care systems around the world, SSI rates vary markedly within surgical categories and are poorly represented by routinely monitored indicator procedures (eg, mastectomy or hernia surgery). Therefore, relying on indicator procedures to estimate the burden of SSI is imprecise and introduces bias as hospitals may take special precautions to achieve lower SSI rates. The most common cause of SSI is *Staphylococcus aureus* (*S. aureus*), as recently confirmed by a Europe-wide point-prevalence study conducted by the European Centre for Disease Prevention and Control (ECDC).

**Objective:**

The primary objective of this study is to determine the overall and procedure-specific incidence of *S. aureus* SSI in Europe. Secondary objectives are the overall and procedure-specific outcomes as well as the economic burden of *S. aureus* SSI in Europe. Explorative objectives are to characterize the composition of the surgical patient population and to estimate the number of patients at risk for *S. aureus* SSI.

**Methods:**

A retrospective, multinational, multicenter cohort study (*Staphylococcus aureus* Surgical Site Infection Multinational Epidemiology in Europe [SALT] study) with a nested case-control part will be conducted. The study will include all surgical procedures at a participating center in order to prevent selection bias and strengthen the understanding of SSI risk by determining the incidence for all common surgical procedures. Data will be assessed in the cohort population, including 150,000 adult patients who underwent any surgical procedure in 2016, and the case-control population. We will match patients establishing *S. aureus* SSI 1:1 with controls from the same center. Data on demographics, surgery, and microbiology will be exported from electronic files. More detailed data will be captured from the case-control population. The SALT study will include 13 major or academic surgical centers in Europe, comprising 3 in France, 4 in Germany, 2 in Italy, 3 in Spain, and 1 in the United Kingdom. Sites were selected using a feasibility questionnaire.

**Results:**

The SALT study is currently recruiting patients. The aim is to complete recruitment in February 2018 and to close the database in September 2018. The final results are expected by the end of 2018.

**Conclusions:**

Results of the SALT study will help to better understand the precise risk of certain procedures. They will also provide insight into the overall and procedure-specific incidence and outcome as well as the economic burden of *S. aureus* SSI in Europe. Findings of the study may help guide the design of clinical trials for *S. aureus* vaccines.

**Trial Registration:**

ClinicalTrials.gov NCT03353532; https://clinicaltrials.gov/ct2/show/NCT03353532 (Archived by WebCite at http://www.webcitation.org/6xAK3gVmO)

## Introduction

### Background

Surgical site infections (SSIs) are among the most common hospital acquired infections and constitute an important quality criterion in health research [1,2]. The incidence of SSIs in certain indicator procedures is the subject of ongoing surveillance efforts in hospitals and health care systems around the world [1,3,4]. However, SSI rates vary markedly within surgical categories and are poorly represented by routinely monitored indicator procedures such as mastectomy, upper limb amputation, or inguinal hernia surgery [4]. Therefore, relying on indicator procedures to estimate the overall burden of SSIs is imprecise and introduces bias as hospitals may take special precautions to achieve lower SSI rates in indicator procedures. *Staphylococcus aureus* (*S. aureus*) is the most common cause of SSIs, as recently confirmed by a Europe-wide point-prevalence study conducted by the European Centre for Disease Prevention and Control (ECDC) [5].

Treatment of *S. aureus* infections is challenging due to the emergence of multi-drug resistant strains such as methicillin-resistant *S. aureus* (MRSA). To better plan future trials on new treatment strategies, knowledge on incidence and risk factors is of utmost importance.

Currently available data suggest that while the overall SSI rate and the proportion of SSIs caused by *S. aureus* vary, the absolute *S. aureus* SSI rate is similar for different procedures. Focusing on *S. aureus* SSIs, we aim to demonstrate that the *S. aureus* SSI rate is independent of the procedure performed and rather a consequence of cutaneous incisions. Such a demonstration would amount to a paradigm shift and allow future prevention studies to proceed differently.

SSIs are associated with poor outcome, prolonged hospitalization, and increased treatment costs [6]. Estimated excess treatment costs range between US $ 1000 to $20,000 [6-10]. Treatment costs depend, among other factors, on the depth of SSI (superficial, deep, or organ space) and the prior surgical procedure. However, the exact treatment costs remain unknown, as previous estimates are based on limited data from single institutions [9], provider networks [11], or highly aggregated data from surveillance programs [7]. Due to this heterogeneous data source, prior cost estimates for Europe show a wide range of results [12].

### Rationale

Active, prospective SSI surveillance lowers SSI rates [13]; therefore, unbiased SSI rates should be determined by retrospective, non-interventional studies. This cohort study will sample a large percentage of the surgical population and thereby generate representative data. For the selection process and documentation of the overall cohort, we will limit data items to those that are generally available in electronic form, thus facilitating the sampling of an adequately sized and complete cohort with comparably low effort. Feasibility analysis of candidate study centers will assess center capability for electronic data provision. If needed, single data items may be waivered during the selection process. In this case, these data items would instead be documented as part of the nested case-control study.

The nested case-control part of this study is necessary to generate data required for outcomes and cost analyses, as most centers will not have electronic records sufficiently detailed to allow this analysis for the whole cohort. The approach avoids bias that has been introduced, for example by relying on reimbursement data to estimate costs [14], and thus describing the SSI price rather than its cost. Therefore, the best possible approach to an accurate, precise cost analysis is manual documentation by on-site medical personnel (eg, study nurses). Documentation must comprise relevant cost drivers of SSI cases and well-matched controls to allow analyses of incremental costs caused by SSI. On-site medical personnel would receive appropriate electronic case report form (eCRF) training prior to commencing documentation. In addition, manual data capturing allows concomitant plausibility checking.

Nesting the case-control study within a cohort ensures generalizability of the case-control results to the respective center as well as the cohort and, by means of a representative cohort, to the overall surgical population in Europe.

### Objectives

#### Primary Objectives

The primary objectives of this study are (1) to determine the overall incidence of *S. aureus* SSIs in Europe; and (2) to determine procedure-specific incidence of *S. aureus* SSIs in Europe.

#### Secondary Objectives

The secondary objectives of this study are (1) to determine the overall outcomes of *S. aureus* surgical SSIs in Europe; (2) to determine the procedure-specific outcomes of *S. aureus* SSIs in Europe; (3) to determine the overall economic burden of *S. aureus* SSIs in Europe; and (4) to determine the procedure-specific economic burden of *S. aureus* SSIs in Europe.

#### Exploratory Objectives

The exploratory objectives of the study are (1) to characterize the composition of the surgical patient population in Europe; (2) to estimate the number of patients at risk for *S. aureus* SSIs; and (3) to estimate the economic burden, including direct treatment and indirect costs, imposed by *S. aureus* SSIs in Europe.

## Methods

### Study Design

This is a retrospective, multinational, multicenter cohort study with a nested case-control. The study includes all surgical procedures at a participating center in order to prevent selection bias and strengthen the understanding of SSI risk by determining incidence for all common surgical procedures. Furthermore, the study will analyze the risk composition of the surgical patient population to enable the calculation of the number of patients at risk in the overall surgical population in Europe.

#### Retrospective Record

**Figure 1:**
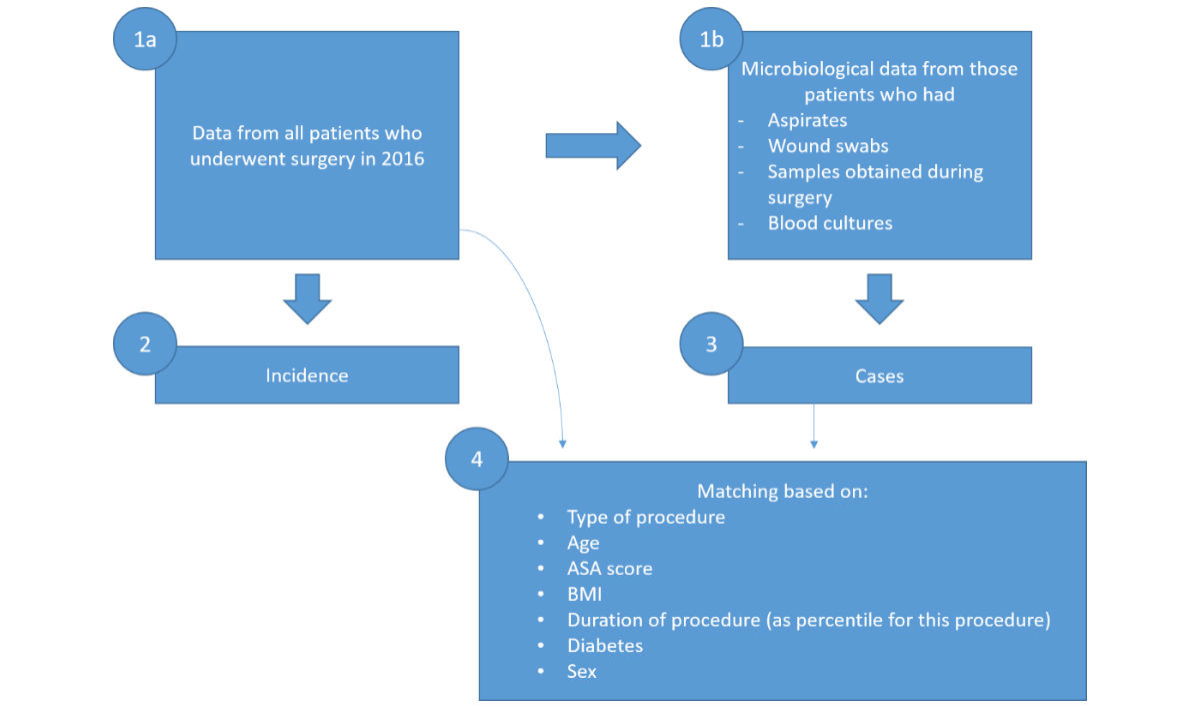
Data assessment and matching procedure. ASA: American Society of Anesthesiologists; BMI: body mass index.

Data from all patients undergoing any surgical procedure—minimal invasive biopsies and eye surgery excluded—will be collected retrospectively. Collection will be performed by surveying electronic health records (EHRs) and databases from all participating sites (Figure 1).

Administrative and microbiological data will be accumulated to identify the target population.

#### Nested Case-Control Design

The nested case-control study generates data for analyzing costs and outcomes. Participants acquiring *S. aureus* SSIs will be documented in an electronic database and matched 1:1 to controls within each center. Criteria for matching are similar epidemiological data and procedure type, but without SSI and are specified in the Data Collection section.

### Study Procedure

#### Populations

Data will be assessed in the cohort and the nested case-control population (Textbox 1).

The following are the matching criteria for the nested case-control population: (1) type of procedure, (2) age, (3) BMI, (4) duration of procedure (as a percentile for this procedure), (5) diabetes, and (6) sex.

#### Study Sites

Approximately 13 surgical centers in Europe will be included in this study. To ensure adequate representation of each type of surgery, only centers with more than 10,000 surgeries will be considered. The distinction between academic and non-academic centers was chosen to ensure broad coverage and prevent selection bias.

Sites interested in participating will be identified through prior publications on SSI, prior SSI study participation, and membership in appropriate European scientific societies, including surgical, microbiological, and infectious diseases societies. Sites will be contacted and selected using a feasibility questionnaire (Multimedia Appendix 1) and procedure.

#### Target Population and Eligibility

All adult patients with a SSI after any surgical procedure—minimal invasive biopsies and eye surgery excluded—in 2016 will be part of our target population. Data from 2015 to 2017 will be collected if not enough data are available in 2015. The inclusion and exclusion criteria are shown in Textbox 2.

**Data assessed in the cohort and nested case-control population.**

- Cohort population
  - Export of electronic file data on demographics
  - Surgical procedure code
  - Duration of procedure
  - American Society of Anesthesiologists score
  - Body mass index
  - Comorbidity International Statistical Classification of Diseases and Related Health Problems codes
  - Wound class of all patients undergoing surgery
- Nested case-control: for patients establishing *S. aureus* surgical site infections (SSI) and 1:1 matched controls from the same center
  - Length of hospitalization
  - Length of intensive care unit (ICU) stay
  - Reason and attribution to SSI
  - Survival at 30 and at 90 days
  - Antibiotic treatments including duration, functional status at admission and at final discharge, necessity for surgical revision, and death attributed to SSI
  - If readmission is necessary, the following will be recorded:
    - Reason and attribution to SSI
    - Length of hospitalization
    - Length of ICU stay
    - Antibiotic treatments and their duration

**Inclusion and exclusion criteria.**

- Inclusion
  - Age 18 years or greater at the time of surgery
- Exclusion
  - Patients undergoing minimal invasive biopsies and eye surgery
  - SSI at the time of surgery
  - Cases with missing data defined as missing completely at random

#### Time Schedule

**Figure 2:**
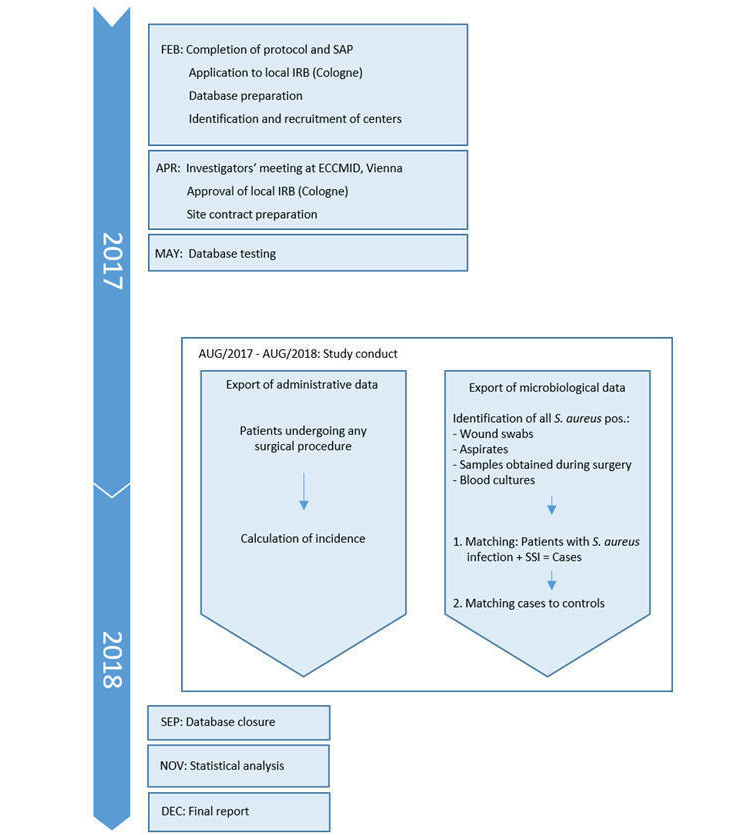
Study timelines. SAP: Statistical Analysis Plan; IRB: institutional review board; ECCMID:European Congress of Clinical Microbiology and Infectious Diseases; SSI: surgical site infections.

The time schedule for the study is shown in Figure 2.

#### Sample Size

We assume a 1.5% incidence rate of *S. aureus* SSI. Incidence rates can be determined with a 95% CI of plus or minus 0.5% by observing 1500 surgical procedures, assuming a mean *S. aureus* SSI rate of 1%. Lower incidence rates would result in smaller 95% CIs with a similar sample size or similar CIs with a smaller sample size. Therefore, at least 15 centers should be included in the analysis, resulting in 90,000 to 150,000 patients observed patients. This will allow us to calculate incidence rates with meaningful precision for all surgical procedures performed on at least 1% to 1.5% of respective patients. To further broaden the scope of this study, 1 or 2 centers per country will be included specializing in types of surgery not traditionally performed at academic surgical centers but highly relevant to the prevention of SSI in otherwise healthy patients (eg, plastic surgery). Assuming a 1.5% incidence rate of *S. aureus* SSI, in a population of 150,000, 2250 cases will be matched to 2250 controls.

#### Data Collection

##### Cohort

The following variables will be collected from EHRs for descriptive purpose only: (1) age by category; (2) sex; (3) BMI; (4) comorbidities (International Statistical Classification of Diseases and Related Health Problems [ICD-10] codes); (5) diabetes; (6) type of procedure; (7) duration of procedure; (8) occurrence of SSI related to observed procedure within 90 days, including date of diagnosis; (9) wound class.

##### Surgical Site Infection Cases

SSI cases will be identified through EHR review in conjunction with microbiological data. The data recorded is shown in Textbox 3.

##### Detection of *S. aureus* Surgical Site Infection

As a first step, we will export the data listed in the Data Collection Cohort section from all patients who underwent surgery in each center in 2016. We expect a minimum of 100,000 cases to be included in the cohort. *S. aureus* SSI that occurred in 2016 will be detected using microbiological data. 

To ensure feasibility, only centers with the capability of electronic microbiologic data export will participate. All patients presenting a *S. aureus* infection from the following will be identified: (1) wound swabs, (2) aspirates, (3) samples obtained during surgery, and (4) blood cultures.

Subsequently, electronic matching with clinical data will be performed in order to determine those patients who underwent a surgical procedure and had an *S. aureus* infection. This approach provides a sensitivity of 100% for identification of proven *S. aureus* SSI cases.

**Data collected for surgical site infection cases.**

- Length of hospitalization
- Length of intensive care unit (ICU) stay
  - Reason for ICU stay; attribution to surgical site infections (SSI)
  - Hours of mechanical ventilation
  - Hemodialysis
- Survival at 30 and 90 days
  In case of death, attribution to SSI
- Necessity for revision surgery
  In case of SSI, attribution to SSI

- Necessity for readmission
  In case of readmission:
  - Reason
  - Attribution to SSI
  - Length of hospitalization
  - Length of ICU stay
    - Hours of mechanical ventilation
    - All antibiotic treatments, including duration and dosage
- All antibiotic treatments, including duration
- Functional status at admission and at final discharge
- Causative pathogens including resistance patterns
- Type of SSI according to European Centre for Disease Prevention and Control criteria

All patients identified will be documented manually. Occurrences of SSI will be verified on a case-by-case following the criteria of the Hospital Acquired (HAI) SSI protocol [1].

###### Superficial Incisional

Infection occurs within 30 days after the operation and involves only skin and subcutaneous tissue of the incision and at least 1 of the following: (1) purulent drainage with or without laboratory confirmation, from the superficial incision; (2) organisms isolated from an aseptically obtained culture of fluid or tissue from the superficial incision; (3) at least 1 sign or symptom of infection (pain or tenderness, localized swelling, redness, or heat and superficial incision is deliberately opened by surgeon, unless incision is culture-negative); and (4) diagnosis of superficial incisional SSI made by a surgeon or attending physician.

###### Deep Incisional

Infection occurs within 30 days after the operation if no implant is left in place or within 1 year if the implant is in place and the infection appears to be related to the operation and infection involves deep soft tissue (eg, fascia, muscle) of the incision and at least 1 of the following: (1) purulent drainage from the deep incision but not from the organ/space component of the surgical site; (2) a deep incision spontaneously dehisces or is deliberately opened by a surgeon when the patient has at least 1 sign or symptom (fever greater than 38° C, localized pain or tenderness, unless incision is culture-negative); (3) an abscess or other evidence of infection involving the deep incision is found on direct examination, during reoperation, or by histopathologic or radiologic examination; or (4) diagnosis of deep incisional SSI made by a surgeon or attending physician.

###### Organ Space

Infection occurs within 30 days after the operation if no implant is left in place or within 1 year if the implant is in place and the infection appears to be related to the operation and infection involves any part of the anatomy (eg, organs and spaces) other than the incision that was opened or manipulated during an operation and at least 1 of the following: (1) purulent drainage from a drain that is placed through a stab wound into the organ/space; (2) organisms isolated from an aseptically obtained culture of fluid or tissue in the organ/space; (3) an abscess or other evidence of infection involving the organ/space that is found on direct examination, during reoperation, or by histopathologic or radiologic examination; or (4) diagnosis of organ/space SSI made by a surgeon or attending physician.

##### Controls

Cases will be matched to controls that received the same procedure but did not develop a SSI. Further variables to be included are (1) age; (2) sex; (3) American Society of Anesthesiologists (ASA) score; (4) BMI; (5) duration of operation (as a percentile); and (6) diabetes. If available, comorbidities other than diabetes and underweight or overweight as well as the Charlson comorbidity index [15] and its components will be included.

### Limitations

As this project is performed at different surgical centers in 5 countries across Europe, procedure and documentation characteristics may vary. Thus, the data set has to be limited to a common set of data frequently reported by all sites.

### Statistical Analysis

Assuming a mean *S. aureus* SSI rate of 1% [5], incidence rates can be determined with a 95% CI of plus or minus 0.5% by observing 1500 surgical procedures. Therefore, at least 15 centers will be included in the analysis, resulting in 90,000 to 150,000 observed patients. Assuming an incidence rate of *S. aureus* SSI of 1.5% in a population of 150,000, 2250 cases will be matched to 2250 controls.

Data will first be analyzed for missing values. We will perform a qualified evaluation of missingness mechanisms for each variable with more than 1% missing values. In case data are missing completely at random, patients with the missing value will be excluded from that respective analysis step (complete record analysis). For values not missing at random, multiple imputation using chained equations will be performed and compared to a complete record analysis for improved robustness of results. We will perform in-depth descriptive statistics of all parameters observed. Country-based and institution-based incidence rates will be determined for each procedure (eg, ventral hernia repair) and each category (eg, vascular surgery). For each incidence rate, the 95% CIs for a binominal proportion will be calculated. Costs will be calculated for both procedures and categories. Accounting for the usually non-normal distribution of costs in medical settings, statistical analyses (eg, CI construction) will be carried out using non-parametric bootstrap procedures. Reported SSI rates and cost will be further stratified by depth of SSI (eg, superficial, deep). Association between major cost drivers and SSI occurrence, as well as overall costs and SSI occurrence, will be determined using bootstrapped t tests.

Multivariable regression analysis will be used to confirm significance of SSI occurrence for overall treatment costs.

#### Primary Analysis

The primary objective of this study is to determine the overall and procedure-specific incidence of *S. aureus* SSI in Europe. The primary analysis will consist of calculating the incidence using 95% CIs.

#### Secondary Analysis

The secondary analysis will consist of determining the overall and procedure-specific outcomes of *S. aureus* surgical site infections in Europe as well as the overall and procedure-specific economic burden of *S. aureus* SSI in Europe. Therefore, patients having established *S. aureus* SSI will be matched 1:1 to controls from the same center based on a propensity score and optimal matching with the following covariates: (1) type of procedure, (2) age, (3) ASA score, (4) BMI, (5) duration of procedure (as percentile for this procedure), (6) diabetes, and (7) sex.

#### Exploratory Analysis

Based on case-control matching, the composition of the surgical patient population in Europe will be characterized and the number of patients at risk for *S. aureus* SSI estimated.

The economic burden, including direct treatment and indirect costs, imposed by *S. aureus* SSI in Europe will be calculated by multiplication of the per-unit price for major cost drivers with the number of units used. The following variables will be included in the cost analysis as major cost drivers: (1) length of hospitalization, (2) length of ICU stay, (3) hours of mechanical ventilation, and (4) number and type of revision surgeries.

#### Further Statistical Evaluation

Distribution of data within groups will be described using count, percentage, or valid n, mean, standard deviation and percentiles (0, 25, 50, 75, and 100) as appropriate. CIs (level 95%) will be calculated to aid interpretation. Sensitivity analyses will include further multiple regression approaches (eg, Poisson regression for rates).

### Data Management

Data will be recorded securely and electronically. The data are the sole property of the sponsor and should not be made available in any form to third parties, except for authorized sponsor’s representatives or appropriate regulatory authorities, without written permission from the sponsor.

The investigator will ensure that all data are entered legibly, completely, accurately, and conform to source documents.

The investigator will review and approve the data; the investigator’s validation serving as attestation of the investigator’s responsibility for ensuring that all data are complete, accurate, and authentic.

All data will be submitted to an automatic control in order to detect missing data, data out of limits, or inconsistent data. The obvious corrections, as spelling mistakes, will be done by the data manager, in accordance with the sponsor, and will be recorded.

All information obtained during the study will be recorded digitally in conformity with the applicable laws and regulations.

### Ethical Standards

The study will be performed in accordance with the all applicable laws and regulations, including the International Conference on Harmonisation (ICH) Guideline for Good Clinical Practice (GCP), the ethical principles that have their origins in the Declaration of Helsinki and applicable privacy laws.

GCP requires that prior to the study onset, the protocol and any other written information regarding this study to be provided to the participant must be approved by an institutional review board (IRB) and/or an independent ethics committee (IEC).

The investigator agrees to allow the IEC/IRB direct access to all relevant documents.

The IEC/IRB must be constituted in accordance with all applicable regulatory requirements. All approvals should be signed by the IEC/IRB chairman or designee and must identify the IEC/IRB name and address, the clinical protocol by title and/or protocol number, the documents received and their version number, and the date approval and/or positive opinion was granted.

The sponsor will provide the investigator with relevant documents that are needed for IEC/IRB review and approval of the study. The sponsor must receive copies of the IEC/IRB approval and any other information that the IEC/IRB has approved for presentation to potential participants.

If the protocol, or any other information that the IEC/IRB has approved is amended during the study, the investigator is responsible for ensuring the IEC/IRB reviews and approves, where applicable, these amended documents. Copies of the IEC/IRB approval of the amended documents and these amended documents must be forwarded to the sponsor.

### Data Confidentiality

The study protocol, documentation, data, and all other information generated by this study will be maintained in a secure manner and will be kept confidential as required by law.

The sponsor will affirm and uphold the principle of the participant’s right to protection against the invasion of privacy. Throughout this study and any subsequent data analyses, all data will be assessed anonymously. No identifiable data (eg, patients name or date of birth) will be assessed. There will also be no use of pseudonyms, which would make a retrospective re-identification of the patient possible. Data collected refers to common treatment modalities in medical care, such that no re-identification of the individual case based on these data will be possible.

The investigator will respect and protect the confidentiality of the subject in all possible ways.

Data access will be limited to study personnel and data will be entered at each site by local study personnel. All information regarding the study, including conduct and results is confidential. No information can be divulged without written consent from the sponsor.

#### Data Handling and Record Keeping

The investigator will be provided with a study file, which should be used to file the protocol, correspondence with the sponsor, and other study-related documents. The investigator must retain the study file for a period of 15 years after the end or premature termination of the study.

The sponsor should inform the center as to when these documents no longer need to be retained. The center should contact the sponsor prior to disposing of any such records.

The study file will be archived according to the procedures applicable in the center. The sponsor should be notified if the Investigator relocates, retires, or for any reason withdraws from the study. The trial records must be transferred to an acceptable designee, such as another investigator, another institution, or to the sponsor.

All documentation pertaining to the study will be kept by the sponsor for at least 15 years after the end or premature termination of the study.

#### Confidentiality, Ownership of Data, and Publication Policy

All information disclosed or provided by the sponsor (or any company/institution acting on their behalf), or produced during the study, including, but not limited to the protocol, the case report forms (CRF), and the results obtained during the course of the study, is confidential prior to the publication of results.

The investigator, and any person under his/her authority, agrees to undertake to keep confidential and not to disclose the information to any third party without the prior written approval of the sponsor. The investigator’s collaborators shall be bound by the same obligation as the investigator. The investigator shall inform collaborators of the confidential nature of the study. The investigator and collaborators shall use the information solely for the purposes of the study, to the exclusion of any use for their own or for a third party’s account.

The submission of this protocol and other necessary documentation to the IEC/IRB and the regulatory authority is expressly permitted, their members having the same obligation of confidentiality.

Statistical analysis and the final report will result in several original articles published in high impact journals.

## Results

The *Staphylococcus aureus* Surgical Site Infection Multinational Epidemiology in Europe (SALT) study is currently recruiting patients. Thirteen surgical centers across Europe have completed the feasibility process and are actively enrolling patients at present. The aim is to complete recruitment in February 2018 and to close the database in September 2018. An investigator meeting is planned for April 2018. The final results are expected by the end of 2018.

## Discussion

### Principal Findings

The SALT study is a cohort study and will sample a large percentage of the surgical population and thereby generate representative data. Use of non-aggregated (ie, patient level) data will allow a precise and differential assessment of SSI burden on health care systems. This study will include all surgical procedures to prevent bias and strengthen the understanding of SSI risk by determining incidence for all common surgical procedures. Furthermore, the study will analyze the risk composition of the surgical patient population to enable calculation of the number of patients at risk in the overall surgical population in Europe. Currently, about 850 million people reside in Europe (depending on the definition of Europe). To improve feasibility, the study is restricted to France, Germany, Great Britain, Italy, and Spain, thus covering about 300 million Europeans from northern, central, and southern European countries for best possible representativeness.

For the selection process and documentation of the overall cohort, data items were limited to those that are generally available in electronic form, thus facilitating the sampling of an adequately sized and complete cohort with comparably low effort. Feasibility analysis of candidate study centers assessed center capability for electronic data provision.

The nested case-control part of this study is necessary to generate data required for outcomes and cost analyses, as most centers do not have electronic records sufficiently detailed to allow this analysis for the entire cohort. The approach prevents bias that has been introduced, for example by relying on reimbursement data to estimate costs [14], and thus describing the SSI price rather than its cost. Therefore, the best possible approach to an accurate precise cost analysis is manual documentation, by on-site medical personnel (eg, study nurses). Documentation must comprise relevant cost drivers of SSI cases and well-matched controls to allow analysis of incremental costs caused by SSI. On-site medical personnel receives appropriate eCRF training prior to commencing documentation. In addition, manual data capturing allows plausibility checking.

Nesting the case-control study within a cohort ensures generalizability of the case-control results to the respective center as well as the cohort and, by means of a representative cohort, to the overall surgical population in Europe.

With SSI rates being the primary endpoint, SSI detection methodology is crucial in this study. Prior studies and surveillance effort, if specifying detection methodology at all, are usually limited to the analysis of administrative code data [14,15]. However, a recent publication demonstrated that using bacteriological data is superior and combining both—administrative and bacteriological data—could be even more advantageous [16]. In this very study, the detection by means of bacteriologic data will result in high sensitivity, specificity, and negative predictive values (96.4%, 91.4%, and 99.9%, respectively) for deep SSI with no significant difference to the use of both types of data.

Cases are matched 1:1 to controls within each center by software provided by the coordinating center. Matching cases and controls within centers addresses the need for risk-adjustment to institutional characteristics and practices. Cases are matched to controls that received the same type of procedure. Individual pair-wise matching will be used, utilizing optimal matching to ensure the overall smallest possible Euclidean distance between cases and controls. Acknowledging the limited utility of using the National Healthcare Safety Network (NHSN) risk index, which only accounts for the existence of certain risk factors but not their composition, a propensity score is determined by logistic regression.

Costs are calculated by multiplication of the per-unit price for major cost drivers with the number of units used. Minor cost drivers, such as drug use, will be disregarded, as length of stay has been repeatedly shown to account for 90% of excess SSI costs [11].

### Limitations

A limitation of this study is the inability to detect late infections occurring post discharge by only analyzing in-patient laboratory data for SSI case identification. Depending on the procedure in question, infections occurring after discharge constitute a notable percentage of SSI. A post discharge surveillance approach will be subject of a future sub study.

### Conclusion

Results of the SALT study will help to better understand the risk of certain procedures. It will allow conclusions on the overall and the procedure-specific outcomes as well as the economic burden of *S. aureus* SSI in Europe. Furthermore, the composition of the surgical patient population in Europe will be characterized and the number of patients at risk for *S. aureus* SSI will be estimated. Findings of the study may help designing clinical trials for *S. aureus* vaccines.

## Appendix

Multimedia Appendix 1 Feasibility questionnaire.

## Abbreviations

ASA: American Society of Anesthesiologists
BMI: body mass index
CRF: case report form
ECDC: European Centre for Disease Prevention and Control
eCRF: electronic case report form
EHR: electronic health record
GCP: good clinical practice
ICD: International Statistical Classification of Diseases and Related Health Problems
ICU: intensive care unit
IEC: independent ethics committee
IRB: institutional review board
*S. aureus*: *Staphylococcus aureus*
SSI: surgical site infections
