# Metadata Correction: Epidemiology of Surgical Site Infections With Staphylococcus aureus in Europe: Protocol for a Retrospective, Multicenter Study

**DOI:** 10.2196/10712

**Published:** 2019-01-07

**Authors:** Sibylle C Mellinghoff, Jörg Janne Vehreschild, Blasius J Liss, Oliver A Cornely

**Affiliations:** 1 Department I of Internal Medicine University Hospital of Cologne University of Cologne Cologne Germany; 2 Department I of Internal Medicine Helios University Hospital Wuppertal Wuppertal Germany; 3 Cluster of Excellence Cellular Stress Responses in Aging-Associated Diseases University of Cologne Cologne Germany

The authors of the paper “Epidemiology of Surgical Site Infections With Staphylococcus aureus in Europe: Protocol for a Retrospective, Multicenter Study” (JMIR Res Protoc 2018;7(3):e63) made a mistake in the final stage of proofreading. In the affiliations list, Dr Liss’s affiliation was incorrectly listed as “Department I of Internal Medicine, University Hospital of Cologne, University of Cologne, Cologne, Germany”. Instead, his affiliation should read “Department I of Internal Medicine, Helios University Hospital Wuppertal, Wuppertal, Germany”.

The correction will appear in the online version of the paper on the JMIR website on January 7, 2019, together with the publication of this correction notice. Because this was made after submission to PubMed, PubMed Central, and other full-text repositories, the corrected article also has been resubmitted to those repositories.

