# Supplementary material for: Epidemiology of Surgical Site Infections With Staphylococcus aureus in Europe: Protocol for a Retrospective, Multicenter Study
Source: JMIR Res Protoc. 2018 Mar 12;7(3):e63. doi: 10.2196/resprot.8177 (PMC5869375; doi:10.2196/resprot.8177)
Supplement: Multimedia Appendix 1 [file resprot_v7i3e63_app1.pdf]

1. Appendix

1.1. Feasibility Questionnaire

Welcome to SALT Feasibility Questionnaire!

Staphyloccoccus Aureus Surgical Site infection muLTinational epidemiology

For determination of the incidence of *Staphylococcus aureus* in surgical site infections (SSI) we designed a multinational, multicenter study. Our aim is to build an academic consortium to realize this project.
In addition to identification of the incidence of *Staphylococcus aureus* SSI, this study should analyze the overall and procedure specific outcome as well as the economic burden of Staphylococcus aureus surgical site infections. We will characterize the composition of the surgical patient population in Europe and estimate the number of patients at risk for Staphylococcus aureus surgical site infections. Furthermore, the economic burden in Europe, including direct and indirect costs, will be estimated.

Statistical analysis and final report will result in one or several scientific articles, mutually published in international journals.

1) How would you classify your institution?
    a. Loccal clinic
    b. Major (non-academical) surgical center
    c. Academical surgical center
    d. Speciality surgical center
2) Does your center keep electronic health records?
    i. Yes
    ii. No
3) Is your center capable of exporting an electronic list of a minimum...
    a. All surgical inpatients – including ICD codes, age, sex
        i. Yes
        ii. No
    b. All inpatient procedures – including ICPM codes and duration of procedure
        i. Yes
        ii. No
4) Is microbiological data available as electronic data?
    i. Yes
    ii. No
5) Is your center capable of exporting a list of all microbiological results?
    i. Yes
    ii. No
6) How many surgeries did your hospital perform in 2015 – or the last year with available data?
    a. < 6.000
    b. 6.000 - 10.000
    c. > 10.000

7) What kind of surgeries are performed at your hospital?

General surgery
Trauma surgery
Orthopedic surgery
Vascular surgery
Cardiothoracic surgery
Neurosurgery
Gynecological surgery
Urological surgery
ENT (Ear Nose and Throat) surgery
Plastic surgery

8) How often does your ethics committee meet?

____

9) Have you served as Principle Investigator for any clinical study before?
   i. Yes
   ii. No

10) Have you served as a Sub-Investigator before?

Thank you for participating in SALT-Feasibility Questionnaire!

1.2. Charlson comorbidity index

Weight index of comorbidities [15]

Conditions	Assigned weights for diseases
Myocardial infarct<br>Congestive heart failure<br>Peripheral vascular disease<br>Cerebrovascular disease<br>Dementia<br>Chronic pulmonary disease<br>Connective tissue disease<br>Ulcer disease<br>Mild liver disease<br>Diabetes	1
Hemiplegia<br>Moderate or severe renal disease<br>Diabetes with end organ damage<br>Any tumor<br>Leukemia<br>Lymphoma	2
Moderate or severe liver disease	3
Metastatic solid tumor<br>AIDS	6

1-year mortality [14]

Weighted index of comorbidity	1-year mortality
0	7%
1-2	21%
2-4	43%
>5	54%

1.3. Declaration of Helsinki (current official version)

WMA

WMA Declaration of Helsinki - Ethical Principles for Medical Research Involving Human Subjects

Adopted by the 18th WMA General Assembly, Helsinki, Finland, June 1964
and amended by the:
29th WMA General Assembly, Tokyo, Japan, October 1975
35th WMA General Assembly, Venice, Italy, October 1983
41st WMA General Assembly, Hong Kong, September 1989
48th WMA General Assembly, Somerset West, Republic of South Africa, October 1996
52nd WMA General Assembly, Edinburgh, Scotland, October 2000
53rd WMA General Assembly, Washington DC, USA, October 2002 (Note of Clarification added)
55th WMA General Assembly, Tokyo, Japan, October 2004 (Note of Clarification added)
59th WMA General Assembly, Seoul, Republic of Korea, October 2008
64th WMA General Assembly, Fortaleza, Brazil, October 2013

Preamble

1. The World Medical Association (WMA) has developed the Declaration of Helsinki as a statement of ethical principles for medical research involving human subjects, including research on identifiable human material and data.

The Declaration is intended to be read as a whole and each of its constituent paragraphs should be applied with consideration of all other relevant paragraphs.

2. Consistent with the mandate of the WMA, the Declaration is addressed primarily to physicians. The WMA encourages others who are involved in medical research involving human subjects to adopt these principles.

General Principles

3. The Declaration of Geneva of the WMA binds the physician with the words,

“The health of my patient will be my first consideration,” and the International Code of Medical Ethics declares that, “A physician shall act in the patient's best interest when providing medical care.”

4. It is the duty of the physician to promote and safeguard the health, well-being and rights of patients, including those who are involved in medical research. The physician's knowledge and conscience are dedicated to the fulfilment of this duty.

5. Medical progress is based on research that ultimately must include studies involving human subjects.

6. The primary purpose of medical research involving human subjects is to understand the causes, development and effects of diseases and improve preventive, diagnostic and therapeutic interventions (methods, procedures and treatments). Even the best proven interventions must be evaluated continually through research for their safety, effectiveness, efficiency, accessibility and quality.

7. Medical research is subject to ethical standards that promote and ensure respect for all human subjects and protect their health and rights.

8. While the primary purpose of medical research is to generate new knowledge, this goal can never take precedence over the rights and interests of individual research subjects.

9. It is the duty of physicians who are involved in medical research to protect the life, health, dignity, integrity, right to self-determination, privacy, and confidentiality of personal information of research subjects. The responsibility for the protection of research subjects must always rest with the physician or other health care professionals and never with the research subjects, even though they have given consent.

10. Physicians must consider the ethical, legal and regulatory norms and standards for research involving human subjects in their own countries as well as applicable international norms and standards. No national or international ethical, legal or regulatory requirement should reduce or eliminate any of the protections for research subjects set forth in this Declaration.

11. Medical research should be conducted in a manner that minimises possible harm to the environment.

12. Medical research involving human subjects must be conducted only by

individuals with the appropriate ethics and scientific education, training and qualifications. Research on patients or healthy volunteers requires the supervision of a competent and appropriately qualified physician or other health care professional.

13. Groups that are underrepresented in medical research should be provided appropriate access to participation in research.

14. Physicians who combine medical research with medical care should involve their patients in research only to the extent that this is justified by its potential preventive, diagnostic or therapeutic value and if the physician has good reason to believe that participation in the research study will not adversely affect the health of the patients who serve as research subjects.

15. Appropriate compensation and treatment for subjects who are harmed as a result of participating in research must be ensured.

Risks, Burdens and Benefits

16. In medical practice and in medical research, most interventions involve risks and burdens.

Medical research involving human subjects may only be conducted if the importance of the objective outweighs the risks and burdens to the research subjects.

17. All medical research involving human subjects must be preceded by careful assessment of predictable risks and burdens to the individuals and groups involved in the research in comparison with foreseeable benefits to them and to other individuals or groups affected by the condition under investigation.

Measures to minimise the risks must be implemented. The risks must be continuously monitored, assessed and documented by the researcher.

18. Physicians may not be involved in a research study involving human subjects unless they are confident that the risks have been adequately assessed and can be satisfactorily managed.

When the risks are found to outweigh the potential benefits or when there is conclusive proof of definitive outcomes, physicians must assess whether to continue, modify or immediately stop the study.

Vulnerable Groups and Individuals

19. Some groups and individuals are particularly vulnerable and may have an increased likelihood of being wronged or of incurring additional harm.

All vulnerable groups and individuals should receive specifically considered protection.

20. Medical research with a vulnerable group is only justified if the research is responsive to the health needs or priorities of this group and the research cannot be carried out in a non-vulnerable group. In addition, this group should stand to benefit from the knowledge, practices or interventions that result from the research.

Scientific Requirements and Research Protocols

21. Medical research involving human subjects must conform to generally accepted scientific principles, be based on a thorough knowledge of the scientific literature, other relevant sources of information, and adequate laboratory and, as appropriate, animal experimentation. The welfare of animals used for research must be respected.

22. The design and performance of each research study involving human subjects must be clearly described and justified in a research protocol.

The protocol should contain a statement of the ethical considerations involved and should indicate how the principles in this Declaration have been addressed. The protocol should include information regarding funding, sponsors, institutional affiliations, potential conflicts of interest, incentives for subjects and information regarding provisions for treating and/or compensating subjects who are harmed as a consequence of participation in the research study.

In clinical trials, the protocol must also describe appropriate arrangements for post-trial provisions.

Research Ethics Committees

23. The research protocol must be submitted for consideration, comment, guidance and approval to the concerned research ethics committee before the study begins. This committee must be transparent in its functioning, must be independent of the researcher, the sponsor and any other undue influence and must be duly qualified. It must take into consideration the laws and regulations of the country or countries in which the research is to be performed as well as applicable international norms and

standards but these must not be allowed to reduce or eliminate any of the protections for research subjects set forth in this Declaration.

The committee must have the right to monitor ongoing studies. The researcher must provide monitoring information to the committee, especially information about any serious adverse events. No amendment to the protocol may be made without consideration and approval by the committee. After the end of the study, the researchers must submit a final report to the committee containing a summary of the study's findings and conclusions.

Privacy and Confidentiality

24. Every precaution must be taken to protect the privacy of research subjects and the confidentiality of their personal information.

Informed Consent

25. Participation by individuals capable of giving informed consent as subjects in medical research must be voluntary. Although it may be appropriate to consult family members or community leaders, no individual capable of giving informed consent may be enrolled in a research study unless he or she freely agrees.

26. In medical research involving human subjects capable of giving informed consent, each potential subject must be adequately informed of the aims, methods, sources of funding, any possible conflicts of interest, institutional affiliations of the researcher, the anticipated benefits and potential risks of the study and the discomfort it may entail, post-study provisions and any other relevant aspects of the study. The potential subject must be informed of the right to refuse to participate in the study or to withdraw consent to participate at any time without reprisal. Special attention should be given to the specific information needs of individual potential subjects as well as to the methods used to deliver the information.

After ensuring that the potential subject has understood the information, the physician or another appropriately qualified individual must then seek the potential subject's freely-given informed consent, preferably in writing. If the consent cannot be expressed in writing, the non-written consent must be formally documented and witnessed.

All medical research subjects should be given the option of being informed about the general outcome and results of the study.

27. When seeking informed consent for participation in a research study the physician must be particularly cautious if the potential subject is in a dependent relationship with the physician or may consent under duress. In such situations the informed consent must be sought by an appropriately qualified individual who is completely independent of this relationship.

28. For a potential research subject who is incapable of giving informed consent, the physician must seek informed consent from the legally authorised representative. These individuals must not be included in a research study that has no likelihood of benefit for them unless it is intended to promote the health of the group represented by the potential subject, the research cannot instead be performed with persons capable of providing informed consent, and the research entails only minimal risk and minimal burden.

29. When a potential research subject who is deemed incapable of giving informed consent is able to give assent to decisions about participation in research, the physician must seek that assent in addition to the consent of the legally authorised representative. The potential subject's dissent should be respected.

30. Research involving subjects who are physically or mentally incapable of giving consent, for example, unconscious patients, may be done only if the physical or mental condition that prevents giving informed consent is a necessary characteristic of the research group. In such circumstances the physician must seek informed consent from the legally authorised representative. If no such representative is available and if the research cannot be delayed, the study may proceed without informed consent provided that the specific reasons for involving subjects with a condition that renders them unable to give informed consent have been stated in the research protocol and the study has been approved by a research ethics committee. Consent to remain in the research must be obtained as soon as possible from the subject or a legally authorised representative.

31. The physician must fully inform the patient which aspects of their care are related to the research. The refusal of a patient to participate in a study or the patient's decision to withdraw from the study must never adversely affect the patient-physician relationship.

32. For medical research using identifiable human material or data, such as research on material or data contained in biobanks or similar repositories, physicians must seek informed consent for its collection, storage and/or reuse. There may be exceptional situations where consent would be impossible or impracticable to obtain

for such research. In such situations the research may be done only after consideration and approval of a research ethics committee.

Use of Placebo

33. The benefits, risks, burdens and effectiveness of a new intervention must be tested against those of the best proven intervention(s), except in the following circumstances:

Where no proven intervention exists, the use of placebo, or no intervention, is acceptable; or

Where for compelling and scientifically sound methodological reasons the use of any intervention less effective than the best proven one, the use of placebo, or no intervention is necessary to determine the efficacy or safety of an intervention

and the patients who receive any intervention less effective than the best proven one, placebo, or no intervention will not be subject to additional risks of serious or irreversible harm as a result of not receiving the best proven intervention.

Extreme care must be taken to avoid abuse of this option.

Post-Trial Provisions

34. In advance of a clinical trial, sponsors, researchers and host country governments should make provisions for post-trial access for all participants who still need an intervention identified as beneficial in the trial. This information must also be disclosed to participants during the informed consent process.

Research Registration and Publication and Dissemination of Results

35. Every research study involving human subjects must be registered in a publicly accessible database before recruitment of the first subject.

36. Researchers, authors, sponsors, editors and publishers all have ethical obligations with regard to the publication and dissemination of the results of research. Researchers have a duty to make publicly available the results of their research on human subjects and are accountable for the completeness and accuracy of their reports. All parties should adhere to accepted guidelines for ethical reporting. Negative and inconclusive as well as positive results must be published or otherwise made

publicly available. Sources of funding, institutional affiliations and conflicts of interest must be declared in the publication. Reports of research not in accordance with the principles of this Declaration should not be accepted for publication.

Unproven Interventions in Clinical Practice

37. In the treatment of an individual patient, where proven interventions do not exist or other known interventions have been ineffective, the physician, after seeking expert advice, with informed consent from the patient or a legally authorised representative, may use an unproven intervention if in the physician's judgement it offers hope of saving life, re-establishing health or alleviating suffering. This intervention should subsequently be made the object of research, designed to evaluate its safety and efficacy. In all cases, new information must be recorded and, where appropriate, made publicly available.

Disclaimer: © 2013 World Medical Association, Inc. All Rights Reserved. All intellectual property rights in the Declaration of Helsinki are vested in the World Medical Association. Previous permissions granted to publish or reproduce otherwise WMA policy don't apply to this version of the Declaration of Helsinki until January 1st, 2014. For further questions, please contact the WMA secretariat at secretariat@wma.net.

© World Medical Association, Inc. - All Rights reserved.
© Asociación médica mundial - Todos los derechos reservados.
© L'Association Médicale Mondiale - Tous droits réservés.
